# Left Atrial High-grade Sarcoma With Focal Osteosarcomatous Differentiation

**DOI:** 10.7759/cureus.7660

**Published:** 2020-04-13

**Authors:** Jessica Napuri, Jose Paz, Pedro Valdes

**Affiliations:** 1 Internal Medicine, Palmetto General Hospital, Hialeah, USA; 2 Cardiology, Palmetto General Hospital, Hialeah, USA

**Keywords:** left atrium, sarcoma, osteosarcomatous, left atrial sarcoma with focal osteosarcomatous differentiation

## Abstract

Approximately 75% of cardiac tumors are benign, and 25% are malignant cardiac tumors. Of these, sarcomas are extremely rare and have been described in isolated case reports. Due to its rarity, there is no published guideline for the management of this pathological entity. We present a case of an 85-year-old female who presented to our hospital with a chief complaint of shortness of breath and pinpointed left-sided chest pain. Computed tomography of the chest showed a filling defect in the left atrium concerning a mass versus thrombus. A transesophageal echocardiogram showed a 4 cm multi-lobular echogenic mass with calcifications in the left atrium likely arising from the pulmonary vein suspicious for malignancy. Cardiovascular surgery department scheduled the patient for surgical debulking/removal via a minimally invasive approach. The specimen was reported to be multi-lobular and was resected in several fragments of tan, fleshy, and somewhat gelatinous appearing tissue in aggregate. Histopathology showed spindle cell malignant neoplasm with small foci of bone and cartilaginous formation, suggestive of osteosarcoma. Expert consultation at John Hopkins reported this to be a high-grade sarcoma with focal osteosarcomatous differentiation. Cardiac synovial sarcomas are less than 0.1% of all primary cardiac tumors reported in the literature. Cardiac synovial sarcomas are not extensively described in literature due to their low incidence and prevalence. Thus, it is important to report cases and follow outcomes. This case reports an extremely rare diagnosis that has been reported in less than seven case reports.

## Introduction

Approximately 75% of cardiac tumors are benign, the most common being myxoma in the left atrium, which requires prompt surgical resection due to the risk of embolization and cardiovascular complications [1]. The remaining 25% are malignant cardiac tumors. Of these, sarcomas are extremely rare and have been described in isolated case reports [2-6]. Cardiac sarcomas have been reported to progress rapidly and cause death through the infiltration of the myocardium by obstructing circulation or by distant metastases [4]. Due to its rarity, there is no published guideline for the management of this pathological entity.

## Case presentation

We present a case of an 85-year-old female with a past medical history of right breast cancer status post mastectomy, hypothyroidism status post thyroid removal, hypertension, hyperlipidemia, and paroxysmal atrial fibrillation who presented to our hospital with a chief complain of shortness of breath and pinpoint left-sided chest pain.

**Figure 1:**
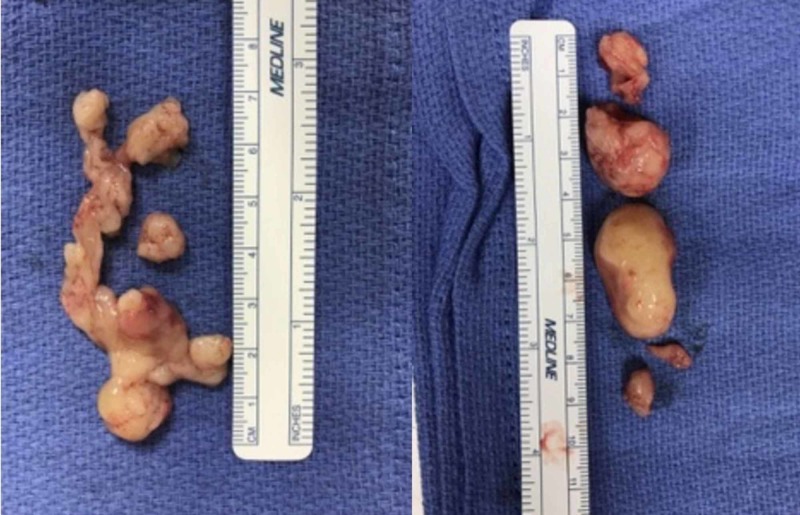
The specimen Several fragments of tan, fleshy and somewhat gelatinous appearing tissue measuring 4.5 x 2.0 x 0.7 cm in aggregate were removed. Pathology received five polypoid tissue fragments ranging from 0.5 up to 3.3 cm and measures in aggregate 5.0 x 3.0 x 2.5 cm.

On the physical exam, the patient was not in any acute distress. She complained of some mild difficulty breathing; however, no accessory muscle usage noted. Lungs were clear to auscultation bilaterally. The respiratory rate was 18 breaths per minute. Three plus pitting edema was noted from the mid-leg shaft to the ankles bilaterally. In the emergency department, an electrocardiogram revealed normal sinus rhythm with occasional premature atrial contractions. Troponin was negative. The outpatient stress test from two weeks prior did not show any signs of ischemia. Computed tomography of the chest showed a filling defect in the left atrium concerning a mass versus thrombus. An immediate bedside contrast transthoracic echocardiogram was performed and confirmed a filling defect with a mass-like lesion adherent to the superior posterior wall, not obstructing the flow of the pulmonary vein. Given the above finding, the patient was scheduled for a transesophageal echocardiogram the following morning which showed a 4 cm multi-lobular echogenic mass with calcifications in the left atrium likely arising from the pulmonary vein suspicious for malignancy. The cardiovascular surgery department requested pan CT which was negative for metastatic malignancy. Due to the patient's co-morbidities, the cardiovascular surgery team recommended left heart catheterization prior to surgery as the patient may also require coronary artery bypass graft (CABG) alongside the excision of the mass. The patient underwent left heart catheterization, which reported no evidence of occlusive coronary artery disease. Cardiovascular surgery department scheduled the patient for surgical debulking/removal via a minimally invasive approach. The specimen was reported to be multi-lobular and was resected in several fragments of tan, fleshy, and somewhat gelatinous appearing tissue in aggregate (Figure 1).

**Figure 2:**
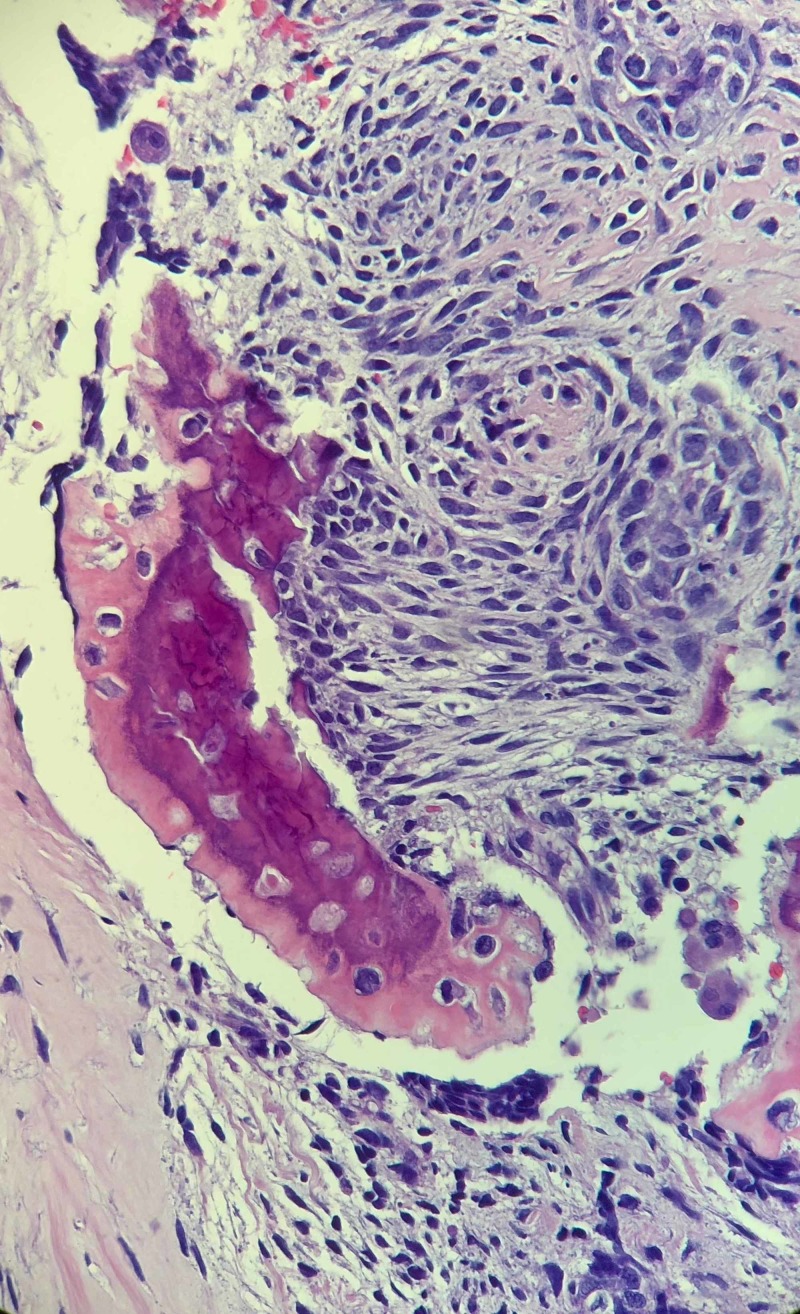
The histopathology findings The overall morphological and immunohistochemical findings argue for primary cardiac synovial sarcoma with focal osteosarcomatous differentiation.

**Figure 3:**
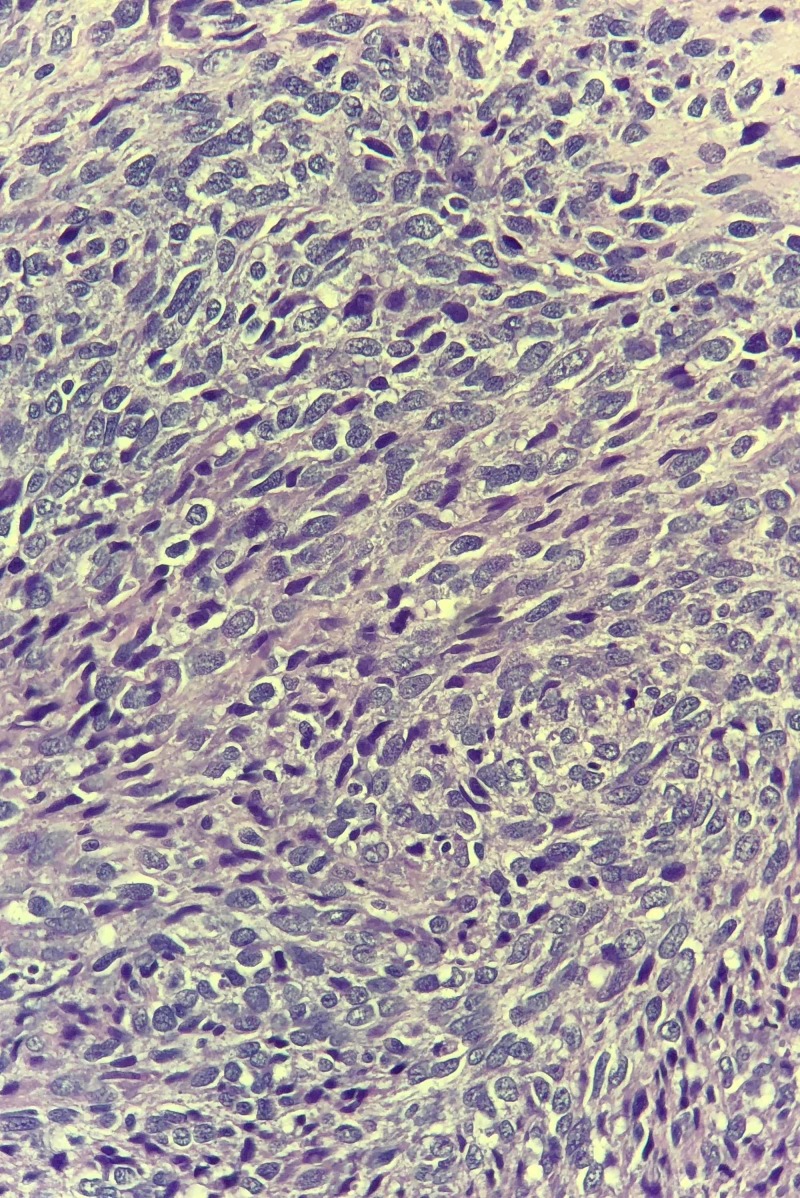
The histopathology findings Immunostains show that the neoplastic cells are positive for panCK (focal), EMA (focal, weak), and negative for S100, SMA, desmin, MART-1, CD34, and CD31.

Histopathology showed spindle cell malignant neoplasm with small foci of bone and cartilaginous formation, suggestive of osteosarcoma. Since this finding is an extremely rare case, the pathology was sent out for expert consultation at John Hopkins, which reported this to be a high-grade sarcoma with focal osteosarcomatous differentiation (Figures 2 and 3).

## Discussion

Twenty-five percent of cardiac tumors are malignant. Of these, sarcomas are extremely rare and have been described in isolated case reports. Synovial sarcomas mainly occur in para-articular soft tissues of the extremities of young adults and adolescents [1, 7]. Cardiac synovial sarcomas are less than 0.1% of all primary cardiac tumors reported in the literature [2, 3]. Typically, they have a male preponderance and are usually right-sided myocardial tumors. However, cardiac synovial sarcomas found in females, on the left side, and in the pericardium have been reported as the rarest of these cases [8]. 

The clinical presentation of these patients with primary synovial sarcomas includes chest pain and signs and symptoms of congestive heart failure. Thus, the presentation is nonspecific and can easily be confused for the presentation of congestive heart failure [9]. These tumors are highly aggressive, proliferate quickly, and have high and rapid mortality after discovery [5]. In approximately one-third of patients, complete surgical resection is possible. Unfortunately, literature reports that even after complete excision, high recurrence rates occur with a reported mean survival rate of 24 months [9]. However, longer survival rates have been reported with multimodality treatment options such as chemotherapy and radiotherapy [10]. Much continues to remain unknown. Currently, there are no published guidelines for the management or treatment of this pathological entity due to its rarity and absence of double-blinded controlled studies. In reporting this case, we hope to enlighten physicians on the rare possibility of primary synovial cardiac tumors and encourage reporting presentation, symptoms, and outcomes of these cases in hopes to aid in defining management and treatment through literature review. 

## Conclusions

Cardia synovial sarcomas are not extensively described in literature due to their low incidence and prevalence. Thus, it is important to report cases and follow outcomes.This case reports an extremely rare diagnosis that has been reported in less than seven case reports. Much remains unknown due to its rarity. 
